# Effectiveness of Continuity of Care Interventions Linking Hospital Care to Primary Healthcare for Patients With Cancer: A Systematic Review and Meta‐Analysis

**DOI:** 10.1002/cam4.71574

**Published:** 2026-02-25

**Authors:** Jiawei Geng, Erxu Xue, Ran Li, Meng Hu, Zhihong Ye, Therese Hesketh

**Affiliations:** ^1^ Centre for Global Health, School of Public Health Zhejiang University School of Medicine Hangzhou Zhejiang China; ^2^ Nursing Department, Sir Run Run Shaw Hospital Zhejiang University School of Medicine Hangzhou Zhejiang China; ^3^ Institute for Global Health University College London London UK; ^4^ Department of Health Management Fuwai Hospital, Chinese Academy of Medical Sciences and Peking Union Medical College Beijing China; ^5^ Faculty of Life Sciences and Medicine King's College London London UK

**Keywords:** cancer, continuity of care, interventions, meta‐analysis, primary health care, quality of care

## Abstract

**Background:**

The growing burden of cancer necessitates continuity of care interventions linking hospital and primary health care (PHC). However, optimal approaches for improving effectiveness and for enhancing care coordination of PHC integration remain unclear.

**Methods:**

A systematic review with meta‐analysis was conducted. Six databases including PubMed, Embase, Web of Science, CINAHL, CENTRAL, and PsycInfo were searched for full texts of randomized or non‐randomized controlled trials (RCTs or nRCTs) from January 2000 to January 2024. Patient‐reported outcomes (quality of care, psychological status), quality of care (adverse events, perceived continuity of care, and satisfaction), healthcare utilization (hospitalization, PHC use, length of stay and emergency visits), and cost evaluation were synthesized. A random‐effects model was used for data analysis, and exploratory subgroup analyses based on intervention characteristics were conducted. Risk of bias was evaluated by using Cochrane Collaboration's Risk of Bias handbooks of RCTs, or Cochrane Risk of Bias Assessment Tool for nRCTs. Intervention strategies aimed at addressing the continuity of care dimensions were synthesized through a deductive approach.

**Results:**

Twenty eight studies from 23 unique interventions were included in the study, all conducted in high‐income countries. Information continuity demonstrated the most effective practice, while management continuity received less emphasis. The overall effect size for quality of life was insignificant (standard mean difference [SMD] 0.01, 95% CI −0.04, 0.05), whereas satisfaction with care was marginally improved in intervention groups (SMD = 0.09, 95% CI 0.01, 0.18). Potential beneficial effects in reduced healthcare utilization and economic savings warrant further study.

**Conclusions:**

Continuity of care interventions by integrating PHC into cancer care is overall not inferior to specialist‐led care. Further efforts to enhance the management continuity dimension of intervention and extend initiatives beyond high‐income countries are warranted.

**Trial Registration:**

PROSPERO registration number CRD42023473024

**Abbreviations**
ACROBAT‐NRSI: Cochrane Risk of Bias Assessment Tool for Non‐Randomized Studies
CENTRAL: Cochrane Central Register of Controlled Trials
CI: confidence intervals
DASS21: Depression, Anxiety, Stress Scale
EORTC QLQ‐C30: European Organization for Research and Treatment
EQ‐5D: Euro QoL‐5 Dimension
GP: general practitioner
HADS: Hospital Anxiety and Depression Scale
IOM: Institute of Medicine
MHI‐5: Mental Health Inventory
nRCTs: non‐randomized controlled trials
PCP: primary healthcare providers
PHC: primary health care
PHQ‐9: Patient Health Questionnaire
PICOS: Population, Intervention, Comparisons, Outcomes, and Types of Study
POMS: Profile of Mood States Questionnaire
PSVQ: Patient Visit‐Specific Questionnaire
QoL: quality of life
RCTs: randomized controlled trials
ROB: risk of bias assessment
SCP: survivorship care plan
SF‐SUNS: Short‐Form Survivor Unmet Needs Survey
SMD: standard mean difference
WHO: World Health Organization

## 1 Introduction

The disease burden of cancer is increasing due to aging and prolonged survival related to advances in diagnosis and treatment [1]. This increases the need for efficient and affordable care for patients with cancer [2, 3]. The dominant specialist care model, common in many countries, is being challenged as cancer care becomes more complex and the long‐term care needs of patients increase, especially in the presence of co‐morbidities [4]. The limitations of specialist cancer care in providing long‐term aftercare highlight the need for different and locally‐appropriate models of cancer care [5].

The World Health Organization (WHO) defines primary health care (PHC) as offering care that is “first‐contact, accessible, continuous, comprehensive and coordinated person‐focused” [6]. Integrating PHC into cancer delivery is viewed as a promising alternative [7]. In 2005, the landmark study of the Institute of Medicine (IOM) highlighted the potential for coordinating between specialists and PHC to address the unmet needs of patients with cancer during the transition from active treatment to aftercare [3, 4, 8]. Such shared care models involve coordination between settings leading to continuity of care with joint involvement of both PHC and specialists [9, 10, 11]. According to the WHO, continuity of care encompasses four domains: interpersonal (caring relationship between care providers and patients), longitudinal (ongoing interaction with the same healthcare providers), informational (availability and effective transfer of information in various episodes), and management (effective collaborations between sectors) continuity [12]. Considering the overlap of the interpersonal and longitudinal continuity [12], we merged these dimensions into what previous studies refer to as relational continuity with a general focus on the patient‐provider relationship during our evidence synthesis [13].

The importance of continuity of care is recognized, but cancer patients frequently experience fragmented care during their care trajectories [14, 15]. Challenges in implementing continuity of care include insufficient medical information transfer, unstable or poor quality of interpersonal relationships between patients and care providers, and suboptimal cross‐sector coordination and communication [16]. Management continuity is integral but hard to achieve, as it requires collaboration across organizational and professional boundaries, relying on the integration of health systems [17, 18].

Despite the widespread interest in engaging PHC providers in cancer care, the effects of continuity of care interventions on cancer patients and healthcare outcomes remain unclear. The only review that summarized evidence of the shared care model under the continuity of care framework, conducted a decade ago, was inconclusive with limited numbers of studies included [11]. A previous review on patients with breast and colorectal cancers provided a narrative synthesis of continuity of care interventions across the cancer care continuum. While most included studies found no difference in outcomes between intervention and control groups, the lack of quantitative synthesis in the review may potentially lead to overlooking specific effectiveness due to insufficient statistical power in individual studies [10]. Considering this, a meta‐analysis is necessary to improve the precision of effect estimates. It can also explore effect modifications on outcomes and identify the potentially effective intervention components. Several reviews have examined the effectiveness of the shared care model compared to usual hospital‐based care [19, 20, 21]. However, the heterogeneity of intervention approaches included has posed challenges in aggregating evidence specific to continuity of care interventions. Recognizing these research gaps, the aim of this review was (1) to evaluate the effectiveness of continuity of care interventions integrating PHC in cancer care on health‐related outcomes and (2) to explore the components of existing interventions to better inform clinical practice.

## 2 Methods

The study was performed by following the PRISMA guideline (Preferred Reporting Items for Systematic Reviews and Meta‐Analyses) [22]. The study protocol was registered in PROSPERO (CRD42023473024). We searched six databases, including PubMed, Embase, Web of Science, CINAHL, Cochrane Central Register of Controlled Trials (CENTRAL), and PsycInfo. Key search terms include “cancer,” “neoplasms,” “shared care,” “continuity of care,” “aftercare,” “follow‐up care,” “transitional care,” “oncology,” “primary health care,” and “trials.” Boolean operators and truncation symbols were used according to database requirements.

### 2.1 Eligibility Criteria

The Population, Intervention, Comparisons, Outcomes, and Types of Study (PICOS) framework was used. The eligibility criteria for the studies are as follows:

1. *Population*: Aged 18 and above diagnosed with any type of cancer at any stage of the cancer care continuum. Studies included patients with co‐morbidities, but patients with cognitive impairment, such as dementia, were excluded.
2. *Intervention*: studies including a focus on the post‐discharge aftercare process and involving care coordination between oncology hospitals (including PHC centres); at least one component identified related to the WHO continuity of care framework.
3. *Comparisons*: Usual care or no utilization of any continuity of care intervention.
4. *Outcomes*: Studies reporting at least one outcome as follows:were involved: patient‐reported outcomes (health‐related quality of life [QoL], psychological status); quality of care (adverse events, perceived continuity of care, satisfaction); healthcare utilization (readmission rate, emergency room admissions, PHC use); and cost evaluation (cost‐effectiveness, cost‐utility, cost‐saving, cost–benefit).
5. *Study design*: Both randomized controlled trials (RCT) and non‐randomized controlled trials (nRCT) with a concurrent or historical comparison group were included. Studies published from January 2000 to 27 January 2024 were included. Study protocols, meeting abstracts, or not full articles were excluded.

### 2.2 Study Selection and Data Extraction

Two reviewers (JWG and EXX) initially independently searched titles and abstracts. Then, the full text was assessed according to the eligibility criteria. Disagreements between the two reviewers were settled by discussion. Finally, we reviewed the reference list of included studies and reviews to identify missing papers. All data were managed by EndNote X9.

We extracted data from the selected studies by: (1) study characteristics (author, year of publication, study setting), (2) participants (number of participants involved, sex, age, cancer type, cancer stage, stage of cancer care continuum), (3) details about the intervention, including the continuity of care dimensions (relational, informational, and management continuity), involved healthcare providers, mobile health use, needs assessment, intervention duration, follow‐up frequency, and (4) study outcomes extracted by different time periods (no more than 3 months, 3–6 months, 6–12 months, and more than 12 months) if outcomes were measured at multiple time points.

### 2.3 Risk of Bias Assessment (ROB)

For the RCT, the risk of bias was assessed according to Cochrane Collaboration's Risk of Bias handbooks of RCTs. The assessed characteristics include method of randomization, the generation of the sequence, blinding of participants and personnel, the report of outcome, and conflict of interest. The risk levels were classified as low, high, and unclear bias.

For the non‐RCT (nRCT), we used Cochrane Risk of Bias Assessment Tool for Non‐Randomized Studies (ACROBAT‐NRSI) for risk assessment. The assessment characteristics assessed include bias to confounding, selection of participants, measurement of intervention, departures from intended intervention, missing data, measurement of outcomes, and selection of the reported results. Bias was classified as low, moderate, and serious. Review Manager 5.4 was used for the presentation of the results.

### 2.4 Strategy for Data Synthesis

Continuous variables including quality of life, psychological outcomes (distress, depression, and anxiety), assessment of continuity of care, and satisfaction with the care were compared based on the differences between the intervention group and control group, or pre‐ and post‐intervention by using standard mean difference (SMD). The 95% confidence intervals (CI) were estimated by Hedge's *g* method [23]. Between‐study heterogeneity was explored using a random‐effects model, pre‐specified for effect size estimation with the pooling method of DerSimonian‐Laird [24].

Between‐study heterogeneity was evaluated by *I* ^2^, *Τ* ^2^, and *χ* ^2^ (Cochrane *Q*). We determined the heterogeneity level by the cut‐off point of 50% as low or high heterogeneity. A funnel plot with Egger and Begger tests was conducted to adjust the potential effect of publication bias on the interpretation of the results. Sensitivity analyses were also conducted by the leave‐one‐out method. To explore the potential effective components, we also conducted subgroup analyses, with the prespecified subgroups involving defined periods of follow‐up (no more than 3 months, 3 to 6 months, 6 to 12 months, and more than 12 months), dimension of continuity of care (all three continuity dimensions or partial), multidisciplinary team (involving different specialized professionals or not), needs assessment (care needs assessment for patients before discharge or not), and any mobile health involvements (yes or no). We also categorized cancer types by main cancers (colorectal, breast, and prostate cancers) or other cancer to explore the potential source of heterogeneity. Economic outcomes were summarized by narrative synthesis.

The R software (version 4.1.2) was used to analyze the data. Two‐tailed *P* value < 0.05 was deemed as significant.

## 3 Results

**FIGURE 1:**
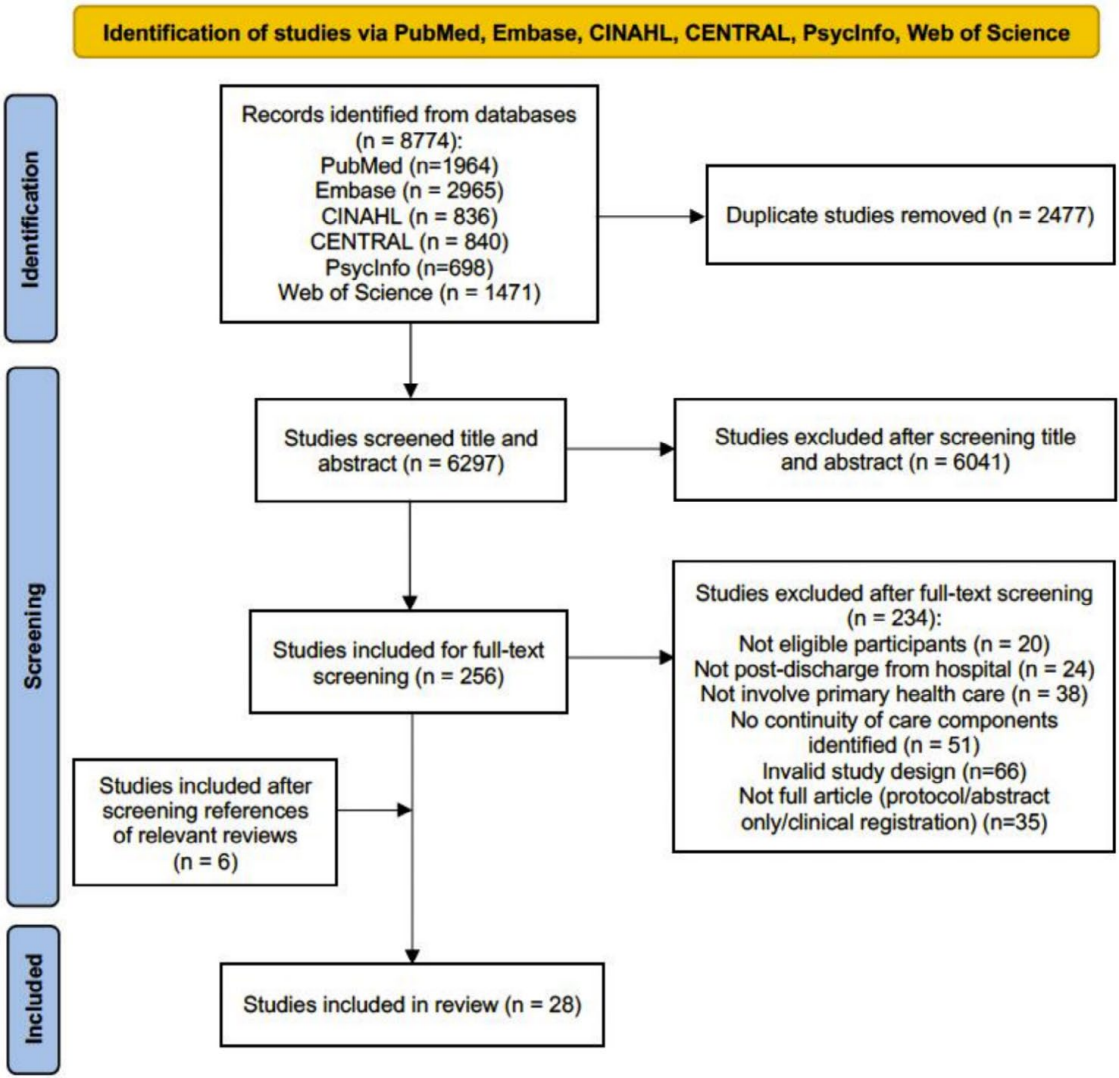
Flow diagram of study selection.

As shown in Figure 1, the search initially yielded 8774 articles. After removing duplicates (*n* = 2477) and those not meeting the selection criteria (*n* = 6275), 22 studies were included. Six studies were further identified from references and relevant reviews. Twenty‐eight articles were included in the review, involving 23 unique interventions. Three interventions reported results in separate articles.

### 3.1 Study and Participants' Characteristics

Of the 23 included interventions, 20 were RCTs [25, 26, 27, 28, 29, 30, 31, 32, 33, 34, 35, 36, 37, 38, 39, 40, 41, 42, 43, 44, 45, 46, 47, 48], one involved a pre‐ and post‐design [49], one used a historical cohort as a comparator [50], and one used a quasi‐experimental design [51]. Studies were published from 2000 to 2024, with 18 published after 2010. The study settings were all in high‐income countries: seven studies in Australia [30, 35, 38, 40, 47, 48, 49], three each from Norway [26, 37, 41, 52], Denmark [27, 28, 45], and the US [42, 43, 44]. Two studies occurred in the United Kingdom [29, 51], Netherlands [31, 32, 33, 34, 46], and Canada [25, 36], with one in Sweden [39] The follow‐up duration ranged from 1 month to 60 months, with a mean follow‐up of 1 year.

A total of 5539 participants were included. The majority of patients were newly diagnosed or shortly after the primary treatment; only two studies focused on the palliative care phase [41, 44, 52]. Types of cancer varied, with breast [36, 39, 42, 46, 49, 51], colorectal [26, 38, 39, 46, 48], and prostate [30, 39] most frequently reported. Seven studies did not identify the cancer type (Table 1; Table S1) [27, 28, 35, 37, 41, 43, 44, 45, 52].

**TABLE 1:** Characteristics of involved studies.

Author	Year	Study design	Setting	Cancer type	Cancer care stage	Number of patients (C/I)	Follow‐up length	Measures of quality of life	Components of interventions^a^
Aubin [25]^b^, ^c^, ^d^	2021	RCT	Canada	Lung cancer	Recent diagnosed	102/104	18 months	/	R1, R3‐R6, I2, I4, M1, M2, M5
Augestad [26]^b^, ^c^, ^d^	2013	RCT	Norway	Colon cancer	Recent surgery, or receiving postsurgical adjuvant chemotherapy	55/55	24 months	EORTC QLQ C‐30, EQ‐5D	R4, R6, I1, I2, I3, I4
Bergholdt [27, 28]^b^, ^c^	2012&2013	RCT	Denmark	Mixed	Newly diagnosed with cancer, diagnosed within the previous 3 month	469/486	14 months	EORTC QLQ‐C30	R1, R2, I2
Canny [29]^b^, ^c^	2022	RCT	UK	Pancreatic or upper gastrointestinal cancer	Newly diagnosed incurable cancers	21/25	12 months	EQ‐5D	R1, I3
Emery [30]^b^, ^c^, ^d^	2017	RCT	Australia	Prostate cancer	Completed treatment for low‐to moderate‐risk prostate cancer with curative intent	43/45	12 months	EPIC	R3, R4, I3
Ezendam [31]& Nicolaije [32]& Rooij [33] & Jeppesen [34] (ROGY care)^c^	2014–2018	RCT	Netherlands	Endometrial and ovarian cancer patients	After initial surgery	173/175	24 months	EORTC QLQ‐C30 & EORTC‐EN24/EORTC‐OV28	I1, I2, I3
Fethney [35]^c^, ^d^	2024	RCT	Australia	Mixed	About to commence a systemic chemotherapy regimen	178/175	Cycle 4 (1 cycle duration mainly 21 days)	EORTC QLQ‐C30	I3, I5, M4, M5
Grunfeld [36]^b^, ^c^, ^d^	2006	RCT	Canada	Breast cancer	After completed adjuvant therapy for early‐stage breast cancer, were disease free	485/483	60 months	SF‐36	R4, R2, R3, M5
Holtedahl [37]^b^	2005	RCT	Norway	Mixed	Finished cancer therapy	50/41	6 months	EORTC QLQ C‐30	R1
Jefford [38]^b^, ^c^, ^d^	2023	RCT	Australia	Colorectal cancer	Completed treatment with curative intent with surgery	76/74	12 months	EORTC QLQ‐C30 & EORTC QLQ‐CR29	R3, R4, I1, I2, I3, M5
Jiwa [49]^b^, ^c^, ^d^	2013	nRCT	Australia	Breast cancer	After surgery	21 (pre‐post)	3 months	SF‐36	R5, I2, M4, M5
Johansson [39]^b^, ^c^, ^d^	2001	RCT	Sweden	Prostate, gastrointestinal, and breast cancer	Newly diagnosed cancer within 3 months	250/260	3 months	/	R5, R6, I1, I2, I3, I5, M3, M6
Johnson [40]^b^, ^c^, ^d^	2015	RCT	Australia	Hematologic, breast, ovarian, or colorectal malignancies	Patients receiving their first round of chemotherapy	46/51	Cycle 6	/	R1, R3, I1, I2, I3, M5
Jordhøy [41, 52]^b^, ^c^, ^d^	2000&2001	RCT	Norway	Mixed	Palliative care	199/235	24 months	EORTC QLQ‐C30	R3, I2, I5, M1, M6
Kvale [42]^c^, ^d^	2016	RCT	America	Breast cancer	Completion of active cancer treatment, excluding hormone treatments and trastuzumab	39/40	3 months	SF‐36	I1, I2, M4
Luker [51]^b^, ^c^	2000	nRCT	UK	Breast cancer	Newly diagnosed with breast cancer, up to 4 weeks from diagnosis	38/38	4 months	/	R1, I2
Mayer [43]^b^, ^c^, ^d^	2016	RCT	America	Mixed	Completed treatment with curative intent	17/20	6 weeks	/	R2, R4, I1, I2, I3, M1
Mitchell [44]^c^, ^d^	2008	RCT	America	Mixed	Palliative care	79/80	9 weeks	AQEL; McGill Quality of Life Questionnaire; Subjective Wellbeing scale	I2, M3, M5
Nielsen [45]^b^, ^c^, ^d^	2003	RCT	Denmark	Mixed	Newly referred to hospital	127/121	6 months	EORTC QLQ‐C30	R1, I1, I2, I3, M3
Perfors [46]^b^, ^c^, ^d^	2022	RCT	Netherlands	Breast, lung, colorectal, gynecologic cancer or melanoma	Newly diagnosed	77/77	9 months	EORTC QLQ‐C30	R1, R5, I2, I4, M3
Rio [50]^b^, ^c^, ^d^	2017	nRCT	Australia	Endometrial cancer	After surgical treatment	73/73	1 month	/	R1, I1, I2, M3, M4
Taylor [47]^c^, ^d^	2019	RCT	Australia	Lymphoma	After treatment completion	30/30	6 months	/	R1, I1, I2
Wattchow [48]^c^, ^d^	2006	RCT	Australia	Colon cancer	At the final postsurgical follow‐up visit, 4–6 weeks after surgery or completion of postsurgical chemotherapy	106/97	24 months	SF‐12	I3, M4

Abbreviations: AQEL, Assessment of Quality of life at the End of Life; C, control; EORTC QLQ C‐30, European Organization for Research and Treatment of Cancer Core Quality of Life questionnaire; EORTC QLQ‐CR29, the colorectal cancer‐specific Quality of Life module of EORTC; EORTC QLQ‐EN24, the endometrial cancer‐specific Quality of Life module of EORTC; EORTC‐OV28, the ovarian cancer‐specific Quality of Life module of EORTC; EPIC, Prostate cancer‐specific quality of life was assessed with the Expanded Prostate Cancer Index Composite; EQ‐5D, EuroQol‐5 Dimension; I, intervention; nRCT, non‐randomized control trial; RCT, randomized control trial; SF‐12, 12‐Item Short Form Survey; SF‐36, 36‐Item Short Form Survey.

^a^ Intervention components by continuity of care dimensions.

^b^ Relational continuity (R): R1: encourage patients to see or contact their primary healthcare providers (PCP); R2: encourage PCP to contact the patients; R3: add additional PCP appointment; R4: replace specialist appointment with patients' PCP; R5: telephone follow‐up by PCP; R6: on‐call system provided by PCP.

^c^ Informational continuity (I): I1: patient self‐management/decision‐support information; I2: supply PCP with summaries related to patients' hospital visit/needs/survivorship care plan; I3: provide PCP relevant management guideline and local resources information; I4: supply the oncology team with patient information results from PCP; I5: organize education sessions for PCP on cancer care.

^d^ Management continuity (M): M1: have clear/organized appointments between sectors; M2: prioritize access to health care providers; M3: establish communication channel between oncology and PCP; M4: smooth referral system; M5: provide updates on care plan and patient progress between sectors; M6: regular supervision of PCP by an oncology team.

### 3.2 Intervention Characteristics

**FIGURE 2:**
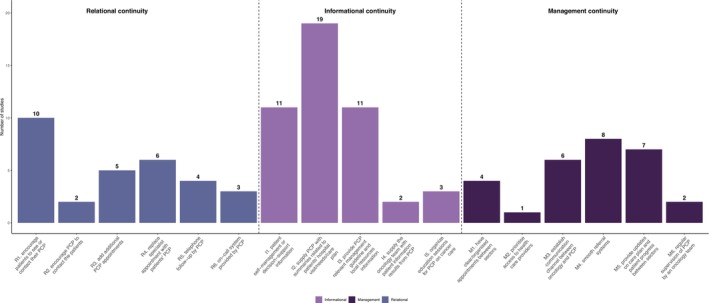
Components of intervention under the conceptual framework of continuity of care. A total of 23 unique studies were included. PCP, primary healthcare providers; SCP, survivorship care plan.

Interventions components were synthesized according to the three dimensions of continuity of care (Figure 2; Table 1). Following the deductive approach, six different elements focusing on relational continuity were identified from eligible studies, including (1) encouraging patients to see or contact their primary healthcare providers (PCP), (2) encouraging PCP to contact patients, (3) adding additional PCP appointment, (4) replacing specialist appointments with patients' PCP, (5) telephone follow‐up by PCP, (6) provision of access to on‐call system provided by PCP. Five elements aimed at informational continuity included (1) providing patient self‐management or decision‐support information, (2) supplying PCP with summaries related to patients' hospital visits or needs or survivorship care plan (SCP), (3) providing PCP relevant management guidelines and local resources information, (4) supplying the oncology team with patient information results from PCP, (5) organizing education sessions for PCP on cancer care. Management continuity components comprised: (1) having clear or organized appointments between sectors, (2) prioritizing access to health care providers, (3) establishing a communication channel between oncology and PCP, (4) a smooth referral system, (5) providing updates on the care plan and patient progress between sectors, (6) regular supervision of PCP by an oncology team.

All studies involved healthcare providers from both PHCs and hospitals, with at least one provider in each setting. Most (*n* = 16) studies additionally involved multidisciplinary team members, such as rehabilitation coordinators, community nurses, nurse navigators, dieticians, psychologists, and general medical practitioners (Table S2) [25, 27, 28, 29, 31, 32, 33, 34, 35, 39, 40, 41, 42, 45, 46, 47, 49, 50, 51, 52]. Most interventions were multifaceted. On average, each intervention applied five components targeted at continuity of care, with a range between one [37] and ten [25]. Information continuity was the most common component with 19 studies supplying PCP with summaries related to patients' hospital visits or needs or SCP [25, 26, 27, 28, 30, 31, 32, 33, 34, 36, 38, 39, 40, 41, 42, 43, 44, 45, 46, 47, 49, 50, 51, 52]. The most used relational continuity and management continuity strategies were encouraging patients to see or contact their PCP (*n* = 10) and streamlining the referral process (*n* = 8), respectively.

### 3.3 Types of Measures and Outcomes

**FIGURE 3:**
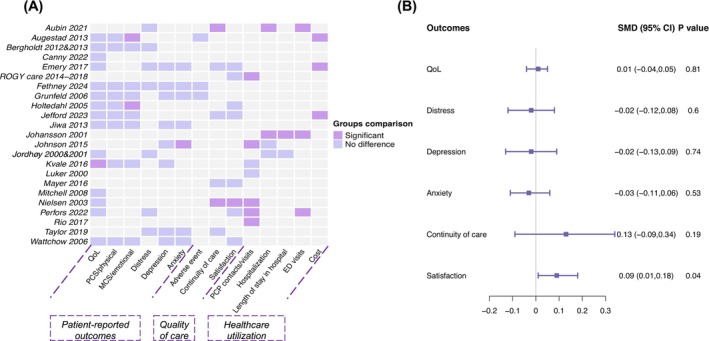
(A) Summary of reported outcomes with or without significant difference between intervention and control or pre and post groups. All significant results favored the intervention group, except Perfors 2022, where significantly more ED visits were reported in the intervention group than the control group. (B) Forest plot of pooled means for perceived QoL, distress, depression, anxiety, continuity of care and satisfaction. For QoL, continuity of care, and satisfaction, positive values favor the intervention over the control; for distress, depression, and anxiety, negative values favor the intervention over the control. ED visits, emergency department visits; MCS, mental component summary; PCP, primary healthcare providers; PCS, physical component summary; QoL, quality of life; SMD, standard mean difference.

To give an overview of results for each study, Figure 3A presented all studied outcomes by intervention. These outcomes included patient‐reported outcomes: (1) QoL (including specific QoL domains, i.e., physical and emotional scales), and psychological status (distress, depression, and anxiety); (2) quality of care (adverse events, continuity of care, and satisfaction); and (3) healthcare utilization outcomes (PCP contacts/visits, hospitalization, length of stay in hospital, ED visit, and cost). Individual studies mostly yielded equal or better outcomes between groups. In Figure 3B, the pooled results from all meta‐analyzed outcomes are presented.

#### 3.3.1 Patient‐Reported Outcomes

##### 3.3.1.1 Quality of Life (QoL)

QoL was used as the primary outcome for most studies. Different QoL measures were used across different studies, with the European Organization for Research and Treatment (EORTC QLQ‐C30) used as the main assessment tool [26, 27, 34, 35, 37, 38, 45, 46, 52], followed by Euro QoL‐5 Dimension (EQ‐5D) and Short Form Survey (Table 1) [42, 48, 49]. Three interventions additionally used specific measures targeted at the particular cancer types [30, 31, 32, 33, 34, 38]. Kvale et al. [42] was the only study that found significantly better overall quality of life in intervention groups compared with controls. As for the specific QoL domains, the emotional role in the intervention group was found to be better than the control group in studies conducted by Augestad et al. [26] and Holtedahl et al. [37] Ten interventions that could provide means and SD were included in the meta‐analysis. For two studies [26, 29] that used multiple scales to measure QoL within the same time frame, we combined the results to mitigate the selection bias.

**FIGURE 4:**
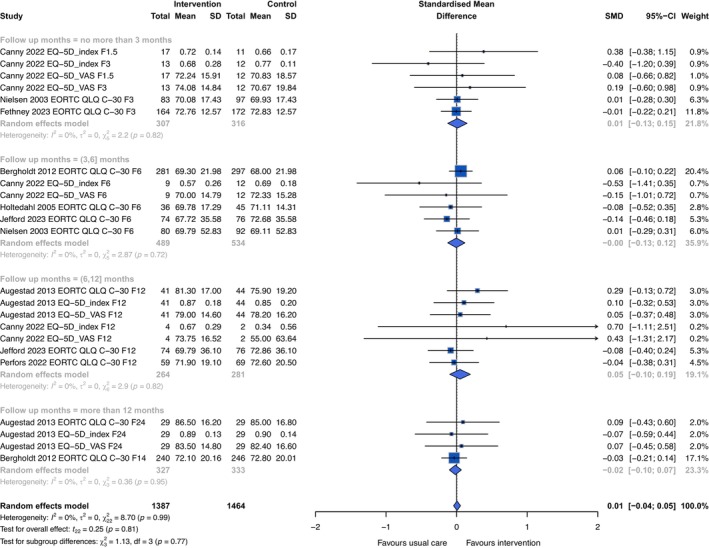
Forest plot of pooled means for quality of life by follow‐up months. EQ‐5D index is the utility scale, and EQ‐5D VAS is the visual analogue scale. The number following F in the study column denotes the length of follow‐up months. Results are presented as standardized mean difference (SMD). SMD indicates the size of the intervention effect in each study relative to the variability observed in that study. The boxes represent point estimates, and the size of the boxes indicates the weight of each study to the overall pooled estimate. Diamonds represent the pooled estimate. A positive value favors the intervention group, while a negative value favors usual care (control group).

The forest plot for overall QoL by follow‐up duration is presented in Figure 4, with a small pooled SMD of 0.01 (95% CI −0.04, 0.05, *I* ^2^ = 0), indicating insignificant results across different time intervals. There was no noticeable change identified in leave‐one‐out sensitivity analyses (Figure S1). We detected no publication bias in the visual inspection of the funnel plot (Figure S2), and both Egger (*p =* 0.693) and Begg (*p =* 0.412) tests were insignificant. Subgroup analyses by intervention characteristics are depicted in Table S3. No significant effect difference was observed in each comparison including stratified studies by different continuity of care dimensions. The pooled effect size on the five main functioning subscales of EORTC QLQ‐C30 was also estimated; however, no difference between interventions and controls was identified.

##### 3.3.1.2 Psychological Status

Distress, anxiety, and depression were the three main psychometric outcomes. Hospital Anxiety and Depression Scale (HADS) was the most commonly used instrument, with studies reporting anxiety and depression using a different scale [30, 35, 40, 48]. One study used Depression, Anxiety, Stress Scale (DASS21), with each of these three outcomes reported individually [47]. Distress was also assessed by the Profile of Mood States Questionnaire (POMS) [27], and Mental Health Inventory (MHI‐5) [46]. Patient Health Questionnaire (PHQ‐9) was used by Kvale et al. [42] to assess depression. As shown in Figure S3, the pooled effect size of the three outcomes did not reach a significant level, with the SMD of −0.02 (95% CI −0.12, 0.08) for distress, −0.02 (95% CI −0.13, 0.09) for depression, and −0.03 (95% CI −0.14, 0.08) for anxiety, respectively, with lower effect sizes indicating better performance in the intervention group.

#### 3.3.2 Quality of Care

##### 3.3.2.1 Continuity of Care

Patients' perceived continuity of care was assessed by six studies with varied instruments. Aubin et al. reported overall better interpersonal and management continuity perceptions by patients in the intervention group [25]. Mayer et al. assessed the care coordination using the adapted Picker Institute Cancer Survey and found no group differences [43]. The remaining four studies evaluated continuity of care by the continuity subscale of Short‐Form Survivor Unmet Needs Survey (SF‐SUNS) [30, 38, 47], were pooled together, with the SMD of 0.13 (95% CI, −0.09, 0.34), indicating no difference between groups (Figure S4).

##### 3.3.2.2 Adverse Events

Fethney et al.'s investigation revealed no adverse event resulting from intervention [35]. The other two studies reported the occurrence of adverse events, such as body weight loss, abdominal pain, and pathologic fracture, and the deterioration in diagnostic indicators including cancer‐suspect lesions, recurrence, hypercalcemia. Both studies reported no difference between the intervention and control groups.

##### 3.3.2.3 Satisfaction

Instruments used to assess satisfaction include Patient Satisfaction Questionnaire [30, 38, 43], Patient Visit‐Specific Questionnaire (PSVQ) [48], and ratings on the general satisfaction with the care [32, 45, 46]. Pooled results yielded an SMD of 0.09 (95% CI, 0.01, 0.18), indicating better performance in the intervention groups compared with the controls (Figure S4).

#### 3.3.3 Healthcare Utilization

Due to the limited number of studies and the heterogeneity in reporting healthcare utilization (categorical or continuous, and different follow‐up lengths), we did not pool the effect size. Two studies reported significantly reduced hospitalization in intervention groups compared with controls [25, 39]. Seven studies measured patients' contact with PCP [32, 33, 40, 42, 45, 46, 50], and five of them observed greater PCP visits for the intervention group than controls [32, 40, 45, 46, 50].

Hospitalization was reported by four studies [25, 39, 41, 51], with two indicating decreased utilization in the intervention group [25, 39]. A comparison of length of stay was conducted in two studies [39, 41], and showed shorter stays only among patients older than 70 years old [39]. Aubin et al. found a modest intervention effect at 3 months after intervention implementation on hospitalization and ED visits. Their findings showed that the number of hospitalizations and ED visits in the intervention group were lower than in the control group (hospitalizations: intervention group 17% vs. control group 29%; ED visits: intervention group 24% vs. control group 36%) [25]. In contrast, Perfors et al. reported increased ED visits in intervention groups compared with controls [46]. Despite most ED visits resulting from oncology‐related issues, the high prevalence of comorbidities in the intervention group might contribute to this.

#### 3.3.4 Cost

Three studies conducted cost‐saving analyses, from either a societal perspective [26] or a health system perspective [30, 38]. All studies replaced certain hospital visits or follow‐up tests with PHC services and found that the cost was lower in intervention groups compared with controls. The shared analytical approach was cost‐minimisation. Augestad et al. undertook the analysis in societal perspective in Norway, including costs such as travel, general practitioner (GP) consultations, specialist outpatient consultations, follow‐up tests, including blood samples, chest x‐ray, ultrasound of liver, CT scans, PET scans, and colonoscopy costs related to sick leave. The study highlighted that the reduction in cost was primarily attributed to the improved coordinated care between hospital and GP, leading to a decrease in hospital visits [26]. Another two studies were conducted in Australia. Emery et al.'s analysis included prostate cancer follow‐up and common chronic disease management, along with relevant health resource utilization. They reported a reduction of US$91 to $554 per patient in the shared care group compared to standard care [30]. In a very recent study, Jefford and colleagues detailed costs across follow‐up care, colorectal cancer investigations, medical services, and other outpatient services. They reported an average cost reduction of US$314 in the intervention group compared to usual care [38]. Overall economic evaluations suggested that the cost reduction was driven by reduced utilization of healthcare services, fewer hospital visits, and timely treatment of other chronic diseases.

### 3.4 Quality Assessment

The summary figures of quality assessment are displayed in Figure S5. Of the 20 RCTs, 12 were deemed to have high ROB [25, 26, 27, 28, 29, 31, 32, 33, 34, 40, 41, 44, 45, 46, 48, 52], while eight [30, 36, 37, 38, 39, 42, 43, 47] had some concerns on ROB. Lack of blinding was the major cause of bias. The three non‐RCTs all demonstrated serious ROB, with confounding bias as the major concern.

## 4 Discussion

This is the latest comprehensive review and meta‐analysis to summarize the evidence on continuity of care interventions linking hospitals to PHC in cancer patients' long‐term care. One of the important contributions of the review is that in addition to effectiveness evaluation, it deductively synthesized intervention strategies for continuity of care. This insight can inform the future design of interventions to enhance care coordination and identify the weakness in such trial development. Pooled results produced generally similar results for patient‐reported outcomes, including QoL, psychological outcomes, and perceived continuity of care between intervention and control groups; and the heterogeneity across studies of these outcomes was low. These findings are consistent with previous reviews on the shared care model, where the insignificant outcomes between intervention and controls were consistently reported [10, 11, 19, 20, 21]. It is hard to disentangle the effective components of the continuity of care intervention, given the multifaceted nature and varying intensity of the involved intervention. However, the limited effect on the intervention may be attributable to partial implementation of the intervention components in real practice, as the collaboration and coordination is hard to measure and may vary in different patients [53]. For example, although in many studies, PCP were supplied by medical summaries or cancer care plans, it is hard to know if the PCP do make use of this information. Similarly, despite some studies using the strategy to establish communication channels between oncology and PCP, the interaction quality between sectors and its impact on improving shared decision‐making process remain unclear. Despite this, there is also a possibility that QoL may not be a sensitive enough indicator for this type of intervention, and the intervention effect may reflect more on patients' care experiences.

Although only 3 studies performed the cost evaluations, it is notable that they consistently reported cost minimization in the intervention group. The potential cost savings associated with utilizing lower‐level healthcare facilities rather than tertiary care were widely recognized as the benefit of shared care intervention. However, there were only three studies including data on cost evaluation [26, 30, 49]. Considering that cost‐driven elements of different interventions varied, further study conducted in different contexts, with more precise documentation of variables is warranted. These three studies all involved substituting specific hospital visits with PHC visits, the major source of cost reduction. Previous evidence suggested that much cancer‐related care, such as the management of long‐term effects, side effects, and psychosocial support does not need specialist care [3, 54].

The use of shared care for cancer based on the continuity of care intervention has been increasing over time in many countries. However, with a limited number of studies included and the varied design in different countries and settings, it is impossible to determine the population that benefited most from the intervention. The complex and varied nature of cancer types and treatment further complicates the picture, which requires further investigation. However, the subgroup analyses reported by Johansson et al. revealed that the older population experienced fewer stays in hospital in the intervention groups than controls [39]. The elderly not only bear a disproportionate burden of cancer but also are vulnerable to other age‐related conditions [55]. The cost evaluation study conducted by Augestad et al. [26], also suggested that increased involvement of PHC might better manage the other chronic diseases of patients, and in turn, contribute to the overall reduction in cost. Therefore, elderly patients might benefit most.

The outcomes measured by the included studies varied, and quality of life was maintained as the main outcome reported by eligible studies. Although included studies contained at least one dimension of continuity of care, only six studies evaluated the outcome of continuity of care. The importance of care continuity in the health care system was less prioritized compared to patient‐centered health‐related outcomes, such as quality of life [56]. Despite this, increasing evidence suggests that patients with cancer who experienced fragmented care are more likely to have a worse prognosis and increased medical expenditure [57, 58]. Future research is needed, preferably including mixed‐method studies, to evaluate patients' experience with continuity of care to enhance the care quality provided along the cancer care continuum.

As for the intervention components addressing different dimensions, we found that information continuity emerged as the most commonly used component in improving outcomes. However, challenges in cross‐sectorial co‐operation between different levels of care settings remained. Recent qualitative studies also highlighted that insufficient personnel coordination is perceived as one of the barriers to integrating PHC into cancer care [59, 60] Further efforts to improve the management continuity are needed.

In the systematic review, purposely linking or re‐connecting the cancer patients with their PCP, who were familiar with their clinical history, emerged as one of the main actionable strategies. As an important component of relationship continuity, it enables the involvement of PCP for patients' cancer care. Besides, this study identified that the most used informational continuity is supplying patients' PCP with summaries related to their cancer‐related medical information, personal care needs, and care plans. However, previous evidence has noted that the process of delivering this summary to PCP should be prioritized over the document itself. Engaging the PCP as a team member for long‐term cancer care is crucial [42]. Practice strategies should target management continuity including clear responsibility between sectors, smooth referral systems, establishing communication channels, and providing progress updates. Of note, effective interventions in management continuity are linked to higher levels of integrated care systems, as shared goals between healthcare sectors necessitate well‐coordinated care [18].

Existing studies were predominantly conducted in high‐income countries with well‐established PHC systems, where GPs or family doctors typically act as gatekeepers. Such healthcare systems provide the necessary resources to implement a shared care model for patients with cancer, who benefit from a pre‐existing relationship [4, 61]. Therefore, findings in these situations might limit the ability for generalization to settings where the role of PHC is weaker. Nonetheless, given the burden of oncology in hospitals, the increasing need for long‐term care, and the financial concern experienced by patients [62, 63], exploring shared care models that offer at least similar reported outcomes, improved satisfaction, and reduced cost is warranted. To enhance the feasibility and acceptability of the model, further research is needed to consider the preferences of patients regarding shared care [64, 65]. Moreover, it is crucial to consider the perspective from the provision side, which includes assessing resources in PHC, understanding PCPs' perspectives on the model, and determining their confidence in providing cancer care before introducing the alternative care model into practice [66].

There are several limitations. First, outcomes reported by the included studies were varied, and results, including assessment of continuity of care and costs, were limited. Second, inconsistent reporting approaches and infrequent occurrence of measurement outcomes hindered the quantification of estimates for several healthcare utilization outcomes. Third, with few studies evaluating continuity of care, we were unable to draw firm conclusions on the effectiveness of the interventions and the effective intervention components. Fourth, only papers published in English were included, potentially biasing outcomes. Fifth, all studies to date were conducted in high‐income countries, thus the generalizability of the evidence is unclear and requires further investigation.

## 5 Conclusion

In summary, the review provided up‐to‐date evidence on the continuity of care interventions based on the shared care model among patients with cancer. At least equivalent effectiveness on patient‐reported outcomes was identified, and the model of care has the potential for higher satisfaction with care. The intervention effect on lower cost and reduced healthcare utilization merits further investigation. Further studies are needed which take into consideration patients' preferences and personal resources. This is of particular importance in low‐income countries where cancer care most needs improvement.

## Author Contributions

**Jiawei Geng:** conceptualization (lead), data curation (lead), formal analysis (lead), methodology (equal), writing – original draft (lead). **Erxu Xue:** data curation (equal), investigation (lead), methodology (equal), validation (lead), writing – review and editing (equal). **Ran Li:** methodology (equal), validation (equal), writing – review and editing (equal). **Meng Hu:** investigation (supporting), methodology (supporting), writing – review and editing (supporting). **Zhihong Ye:** methodology (supporting), writing – review and editing (supporting). **Therese Hesketh:** conceptualization (lead), data curation (lead), methodology (lead), supervision (lead), writing – review and editing (lead).

## Funding

T.H. is supported by National Natural Science of China (72150710552). The funders did not participate in the study's design and implementation, data collection, management, analysis, or interpretation, and were also not involved in the preparation, review, or approval of the manuscript, nor in the decision to submit it for publication.

## Ethics Statement

The authors have nothing to report.

## Consent

The authors have nothing to report.

## Conflicts of Interest

The authors declare no conflicts of interest.

## Supporting information


**Table S1.** Detailed study characteristics.
**Table S2.** Continuity of care dimensions in the intervention group.
**Table S3.** Subgroup analysis on the quality of life.
**Figure S1.** Sensitivity analysis by the leave‐one‐out method on quality of life.
**Figure S2.** Funnel plot assessing publication bias for the quality‐of‐life outcomes.
**Figure S3.** Forest plot of pooled means for perceived (A) continuity of care; (B) satisfaction. Number following F in the study column denotes the length of follow‐up months.
**Figure S4.** Forest plot of pooled means for (A) distress; (B) depression; (C) anxiety. Number following F in the study column denotes the length of follow‐up months.
**Figure S5.** Results of risk of bias presented by each study for (A) randomize control trials; (B) non‐randomized control trials; presented as percentage across all included studies of (C) randomize control trials, and (D) non‐randomized control trial.

**File S1.** PRISMA checklist.

## Data Availability

Data are available upon reasonable request from the corresponding author.
