# Supplementary material for: Effectiveness of Continuity of Care Interventions Linking Hospital Care to Primary Healthcare for Patients With Cancer: A Systematic Review and Meta‐Analysis
Source: Cancer Med. 2026 Feb 25;15(3):e71574. doi: 10.1002/cam4.71574 (PMC12935766; doi:10.1002/cam4.71574)
Supplement: Supplementary file 1 — Table S1. Detailed study characteristics. Table S2. Continuity of care dimensions in the intervention group. Table S3. Subgroup analysis on the quality of life. Figure S1. Sensitivity analysis by the leave‐one‐out method on quality of life. Figure S2. Funnel plot assessing publication bias for the quality‐of‐life outcomes. Figure S3. Forest plot of pooled means for perceived (A) continuity of care; (B) satisfaction. Number following F in the study column denotes the length of follow‐up months. Figure S4. Forest plot of pooled means for (A) distress; (B) depression; (C) anxiety. Number following F in the study column denotes the length of follow‐up months. Figure S5. Results of risk of bias presented by each study for (A) randomize control trials; (B) non‐randomized control trials; presented as percentage across all included studies of (C) randomize control trials, and (D) non‐randomized control trial. [file CAM4-15-e71574-s002.docx]

**Supplementary Tables and Figures**

**Table S1.** Detailed study characteristics.

**Table S2.** Continuity of care dimensions in the intervention group.

**Table S3.** Subgroup analysis on the quality of life.

**Figure S1.** Sensitivity analysis by the leave-one-out method on quality of life.

**Figure S2.** Funnel plot assessing publication bias for the quality-of-life outcomes.

**Figure S3.** Forest plot of pooled means for perceived **(A)** continuity of care; **(B)** satisfaction. Number following F in the study column denotes the length of follow-up months.

**Figure S4.** Forest plot of pooled means for **(A)** distress; **(B)** depression; **(C)** anxiety. Number following F in the study column denotes the length of follow-up months.

**Figure S5.** Results of risk of bias presented by each study for **(A)** randomize control trials; **(B)** non-randomized control trials; presented as percentage across all included studies of **(C)** randomize control trials, and **(D)** non-randomized control trial.

**Table S1.** Detailed study characteristics.

| **Author, year** | **Study objectives** | **Female, number (percentage)** | **Age, mean (SD)** | **Inclusion criteria** | **Exclusion criteria** | **Follow-up interval** |
| --- | --- | --- | --- | --- | --- | --- |
| Aubin, 2021 | To improve continuity of care and interprofessional collaboration, as perceived by patients with lung cancer and their FPs. | C: 44 (43.1) I: 41.3 (64.6) | C: 64.6 (8.4) I: 64.2 (9) | 1) a recent diagnosis of nonsurgical lung cancer (85% of all cases); These patients are followed by a single team, thus facilitating the implementation of the intervention. The 5-year survival rate is approximately 15%; nonetheless, this time frame allowed us to capture patient perceptions at different phases of their disease trajectory; 2) having a family physician (FP); 3) having at least a 3-month estimated life expectancy. | / | Every 3 months |
| Augestad, 2013 | 1) To compare patients’ quality of life and costs of follow-up by their local GP or at the surgical outpatient clinic.; 2) To test whether the incidence of serious clinical events (SCE) would be similar for patients followed up by their GPs or hospital surgeons, and no delay in detection of relapse and the same frequency of SCEs as controls. | C: 22 (40) I: 23 (41.8) | C: 66.7 (7.3) I: 64 (8.7) | 1) age less than 75 years with recent surgery for colon cancer at Dukes’ stage A, B or C; 2) Patients receiving postsurgical adjuvant chemotherapy (some Dukes’ B and all Dukes’ C) were also eligible. | 1) older than 75 years; 2) patients belonging to healthcare trust not participating in the trial; 3) those not able to provide informed consent and cancer stage Dukes’ D. | Every 3 months |
| Bergholdt,2012&2013 | To test the hypothesis that a multimodal intervention giving the general practitioner (GP) enhanced role in cancer rehabilitation improves patients’ quality of life and psychological distress.  **(Bergholdt, 2012)** To improve general practitioner's (GPs) involvement in cancer rehabilitation. **(Bergholdt, 2013)** | C: 335 (71.4) I: 353 (72.6) | C: 62.8 (17.25) I: 62.2 (15) | 1) aged ≥18 years; 2) newly diagnosed with cancer and admitted to Vejle Hospital between 12 May 2008 and 28 February 2009; 3) listed with a GP | 1) patients with carcinoma in situ or non-melanoma skin cancer | On month 6 month, and 14 |
| Canny,2022 | 1) To evaluate the feasibility and acceptability of an early ACP (advanced care planning) intervention in primary health care designed to trigger proactive discussions between patients and their GP leading to a documented advanced care plan in the Scottish primary care electronic record; 2) To empower and encourage patients and their family/carers to engage in an ACP process soon after starting palliative oncology treatment. | C: 7 (33) I: 8 (32) | C: 64 (8) I: 65 (9) | 1) aged ≥18 years; 2) staring palliative treatment for newly diagnosed incurable pancreatic or upper gastrointestinal (esophageal or gastric) cancer at a Scottish regional cancer centre. | 1) People who were unfit for oncology treatment; 2) chose supportive care, had another life-limiting condition; 3) with cognitive impairment that precluded informed consent, communication by telephone, questionnaire completion, or interview. | On week 6, 12, 24, and 48 |
| Emery,2016 | To test the feasibility and efficacy of a multifaceted model of shared care for men after completion of treatment for prostate cancer. | / | C: 65.8 (8.2) I: 67.4 (7.0) | 1) men had completed surgery and/or radiotherapy with curative intent for prostate cancer within the previous 8 weeks; 2) were able to read and write English and had a GP who agreed to participate; 3) Men receiving neoadjuvant ADT with radiation treatment were eligible. | 1) prostate cancer with high-risk features (cT3; PSA ≥20 or Gleason score ≥8); 2) men on androgen deprivation therapy (ADT) after completion of radiotherapy; 3) metastatic disease or treatment with palliative intent; 4) severe cognitive or psychiatric disorder | At 3-, 6- and 12-month |
| Ezendam, 2014 & Nicolaije, 2015 & Rooij 2017 & Jeppesen 2018 (ROGY care) | To assess the effect of sending a survivorship care plan (SCP) to primary care physicians (PCP) on the communication of the PCP with the medical specialist and the patient and to describe PCPs' opinion regarding the SCP | 100% | C: 67.7 (8.8) I: 67.1 (9.1) | 1) age ≥ 18 years (no upper age limit); 2) Diagnosed with endometrial or ovarian cancer | 1) Patients with borderline ovarian cancer; 2) Patients undergoing palliative care; 3) Patients who are not able to complete a Dutch questionnaire | All measures will be collected at the beginning of the trial, and at 6, 12, 18 and 24 months after diagnosis. |
| Fethney,2023 | To determine whether the intervention reduced the number of unplanned hospital presentations by systemic chemotherapy outpatients; and improved systemic chemotherapy outpatients’ physical and psychosocial health outcomes. | C: 109 (61.9) I: 114 (67.1) | C: 59.4 (13.6) I: 59.3 (12.7) | 1) Solid cancer tumour; 2) over 18 years of age; 3) starting the first cycle as an outpatient; 4) fully aware of cancer diagnosis and living within the relevant health district. | 1) being unable to give informed consent; 2) being treated for haematological malignancy; 3) receiving oral chemotherapy, receiving concurrent radiotherapy; 4) not understanding written or spoken English. | Cycle 1, 2, 3, 4 |
| Grunfeld,2006 | To test if follow-up by the patient’s family physician is a safe and acceptable alternative to specialist follow-up. | 100% | C: 60.9 (15) I: 60.9 (16) | 1) women with early-stage breast cancer who had completed adjuvant chemotherapy, radiotherapy, or both at least 3 months previously; 2) who were disease free; 3) and who were between 9 and 15 months after diagnosis; 4) Patients may have continued receiving adjuvant hormonal therapy. | 1) Patients were excluded if they had persistent complications of primary treatment; 2) were unable to comply with the study protocol including completing questionnaires; 3) were previously enrolled in a study that required continued specialist follow-up; 4) were actively observed at a cancer center for another primary cancer. | 6, 12, 18, 24, 36, 48, and 60 months |
| Holtedahl,2005 | To investigate whether increased contact with the patient's general practitioner (GP) soon after cancer treatment can increase patient quality of life. | C:32 (64) I: 19 (46) | C: 63 (NR) I: 61 (NR) | 1) ≥18 years of age or older, 2) living in the Troms municipality in Northern Norway and 3) diagnosed with primary pr replasing cancer during a 1-year period from 1 October 1999 to 30 September 2000. | 1) less than 6 months expected lifetime, mental insufficiency or psychological imbalance as judged by the patient's doctor -in-charge. 2) Pre-cancerous conditions, like basalioma and in situ cervical cancer were not included. Replace was defined as a new tumour revealed at least 2 weeks after ending therapy. | on month 6 |
| Jefford,2023 | To examine shared oncologist and general practitioner (GP) follow-up for survivors of colorectal cancer (CRC). SCORE aimed to show that shared care (SC) was noninferior to usual care (UC) on the EORTC QLQ-C30 Global Health Status/Quality of Life (GHQ-QoL) scale to 12 months. | C: 31 (41) I: 28 (38) | C: 63 (4.5) I: 64 (5.5) | 1) had histologically confirmed diagnosis of colon or rectal cancer; 2) had stage I–III disease; 3) completed treatment with curative intent with surgery, with or without radiation, and with or without chemotherapy, within two months;  4) were over the age of 18 years; were able to understand English, and had a GP willing to participate in the study. | 1) cognitive or psychological difficulties that would preclude participation; 2) too unwell to participate; 3) prior cancer, other than non-melanoma skin cancer, and if the person’s GP was already participating in the study (to avoid contamination). | On month 6 and 12 |
| Jiwa,2013 | To test the feasibility of arranging targeted therapeutic action initiated by GPs on the recommendations of a multidisciplinary team based in primary care. | 100% | 55 (5.75) | listed for visits to a hospital-based surgical follow-up clinic patients were able to nominate a GP who also consented to participate in the study | / | On month 3 |
| Johansson,2001 | 1) To evaluate the effect of intensified primary healthcare services on newly diagnosed cancer patients' utilisation of specialist care after hospital discharge and during periods of radiation and chemotherapy, in comparison to patients in standard care; 2) To investigate if such an effect is modified by the patient's age, after adjustment for weight loss and the level of psychological distress. | C: 111 (56) I: 128 (59) | C: 63.3 (13.2) I: 63 (12.5) | Newly diagnosed with prostate cancer, or gastrointestinal cancer (GI cancer=colorectal or gastric cancer); Breast cancer patients were included in a period ranging from the mammography at which their malignancy was first detected to 3 months after diagnosis. 2. Patients with GI or prostate cancer were included as soon as possible after the confirmation of their diagnosis, but no later than 3 months from diagnosis. | 1) a need for constant hospital care (Karnofsky performance status (KPS) <40); 2) an earlier cancer diagnosis; 3) inability to communicate in Swedish 4) participation in an ongoing randomised trial for patients with localised prostate cancer. 5) patients who were shown to have a benign breast tumour. | On month 3 |
| Johnson,2015 | To test if the intervention would improve patients treated with chemotherapy have higher levels of emotional well-being and empowerment, lower levels of anxiety and distress, and fewer adverse effects compared with people receiving standard care. | C: 41 (89.1) I: 42 (82.4) | C: 56.5 I: 53.2 | Four patient streams were recruited: (1) patients with non-Hodgkin lymphoma receiving cyclophosphamide, vincristine, doxorubicin, and oral prednisolone; (2) women with stages I and II breast cancer offered four to six cycles of adjuvant chemotherapy; (3) women with stages I, II (a-c) and III (a-c) ovarian cancer who had undergone surgical debulking and were offered 6 to 8 cycles of adjuvant chemotherapy with carboplatin and paclitaxel; and (4) patients with cancer of the colon, Dukes B or C, who had undergone surgical resection and offered 6 months chemotherapy with infusion fluorouracil, leucovorin, and oxaliplatin; fluorouracil/leucovorin; or capecitabine. | / | Baseline, mid-study, and after cycle 6 (before, in the middle of, and on completion of chemotherapy) |
| Jordhøy,2000&2001 | To assess the impact of comprehensive palliative care on patients' quality of life. | C: 101 (51) I: 103 (44) | C: 69 (14) I: 70 (13) | 1) Incurable malignant disease; 2) life expectancy of 2–9 months (estimated at referral); 3) and age older than 18 years | 1) Haematological malignant disorders other than lymphomas participation in other trials with health-related quality of life as an outcome. | Every month till death |
| Kvale,2016 | To promote survivor activation and self-management of survivorship health issues as patients transition from active treatment to follow-up care. | 100% | C: 59.51 (11.96) I: 57.23 (9.15) | 1) age ≥19 years; 2) diagnosed with nonmetastatic breast cancer (AJCC TNM stage 0-IIIb); and 3) within 1 year of completing active cancer treatment. | 1) excluding hormone treatments and trastuzumab; 2) the most frequent cause of exclusion being outside of the timing recruitment window (within 1 year of the completion of active treatment). | Baseline, 3 months |
| Luker,2000 | To determine if women with breast cancer were more likely to visit the PHCT for information if the PHCT (primary health care team) had been provided with treatment-specific information, in the form of information cards, from the hospital-based specialist service. | 100% | C: 56.1 (14.2) I: 56.1 (10.2) | 1) women who were newly diagnosed with breast cancer (up to 4 weeks from diagnosis); 2) were aware of their diagnosis and had no previous history of breast cancer. | / | on month 4 |
| Mayer,2016 | 1) To determine feasibility of the intervention and study measures, and to compare the change in confidence in knowledge of future survivorship care needs between survivors and their PCP from baseline to the six-week follow-up; 2) To compare changes from baseline to the sixweek follow-up in measures of PCP visit completion; cancer care coordination; and survivor concerns, expectations, and satisfaction with care. | C: 10 (58.8) I: 12 (63.2) | C: 55.6 (11.3) I: 57.7 (10.9) | **Patient:** 1) being aged 21 years or older; 2) able to read and speak English; 3) diagnosed with an adult onset cancer; 4) having completed treatment with curative intent within four to six weeks; 5) having a designated PCP. **PCP：**1) having a survivor enrolled in this study; 2) a willingness to complete the study measures; 3) being able to have a visit with the survivor within six weeks of SCP receipt if the patient was randomized to the SCP plus PCP arm. | 1) Having metastatic cancer | Baseline, 6 weeks |
| Mitchell,2008 | To test compared with normal care, if case conferences between GPs and specialist teams: 1) improve patient outcomes in terms of QoL, physical and psychosocial parameters and 2) reduce the strain of caring for the primary carers of palliative care patients. | C: 79 (40.5) I: 39 (48.8) | C: 65.2 (13.75) I: 71.8 (13) | 1) have a life expectancy of at least 1 month; 2) were capable of giving informed consent; 3) their GPs and their principal carer (if present) both gave permission to approach the patient. | 1) less than 1 month, inability to give informed consent; 2) being too ill to participate in an interview of approximately 15 minutes duration. | At baseline, then 1, 3-, 5-, 7- and 9-week post- case conference (intervention) or recruitment (control). |
| Nielsen,2003 | To determine the effect of a shared care programme on the attitudes of newly referred cancer patients towards the healthcare system and their health-related quality of life and performance status, and to assess patients' reports on contacts with their GP. | C: 87 (68.5) I: 72 (59.5) | 18-70+ (presented as categorical variable) | 1) Informed consent; 18 years and over;  2) Danish citizens, able to understand and speak Danish; Mentally able to cooperate; Newly referred and diagnosed cancer patients who were scheduled for treatment or attendance for control at the Department of Oncology during a given period; Patients with recurrent disease could be included if their last visit took place later than 3 years previously. | 1) Short palliative radiation therapy without subsequent check-up; 2) Patients enrolled in a special ambulatory with doctors from other departments (dermatologists and otologists); Patients with a diagnosis of lymphoma (this group was transferred to another hospital during the inclusion period); 3) Patient not listed with a GP, health insurance group II patients (patient co-payment, 2–3% of the Danish population) | 3, and 6 months |
| Perfors,2022 | To test the effects of the full GRIP intervention in the year after cancer diagnosis on patient satisfaction, healthcare utilisation, quality of life, mental health and self-efficacy (component of patient empowerment), for patients treated with curative intent. | C: 58 (75.3) I: 57 (74) | C: 59.3 (12.2) I: 61.8 (11.4) | 1) aged ≥18 years; 2) newly diagnosed with either breast, colorectal, gynaecological, lung cancer or melanoma, and 3) scheduled for treatment with curative intent. | 1) in case they were unable to fill in questionnaires, 2) had a major psychiatric disease or personality disorder, 3) already started cancer treatment or 4) if the patient’s GP worked outside the study area or did not agree to participate. | Baseline, on 2 weeks, 12 weeks, 26 weeks, 38 weeks, and 52 weeks |
| Rio,2017 | To develop a GP model of follow-up care after surgical treatment with early endometrial cancer that provided comprehensive clinical handover to a woman's GP and was acceptable to women and GPs. | 100% | NA | 1) early endometrial cancer (FIGO Stages 1 A and B Grades 1 and 2); 2) having a regular GP was confirmed at the hospital multidisciplinary tumour board (MDT) meetings | / | 1 month |
| Taylor,2019 | To use a pragmatic randomised controlled trial (RCT) design to develop and evaluate an evidence-based nurse-led lymphoma survivorship model of care intervention. | C: 18 (60) I: 8 (27) | 18-86 (presented as categorical variable) | 1) A confirmed diagnosis of HL or NHL; 2) completed first-line curative intent chemotherapy or second-line curative intent autologous stem cell transplant within the previous three months; 3) no evidence of lymphoma disease on mid-treatment and/or post-treatment PET scan; 4) over 18 years of age; 5) and ability to understand and read English. | 1) no chemotherapy; 2) treatment or follow-up at another hospital; 3) cognitive or diagnosed acute mental health condition precluding informed consent; 4) diagnosis of a secondary cancer or other medical condition requiring treatment. | 3, and 6 months |
| Wattchow,2006 | 1) To determine whether, among these patients, the setting of follow-up impacts on our primary outcomes: quality of life, psychological well-being and satisfaction with care; 2) To compare the recorded were the follow-up procedures undertaken in the two arms, and data on recurrences and death. | C: 49 (46.2) I: 37 (34.9) | presented as categorical variable from less than 60 to 80+ | 1) surgery for colon cancer (including rectosigmoid) with histological grade Dukes stage A, B or C (cases of disseminated cancer were excluded); 2) completion of postsurgical chemotherapy (principally Dukes Stage C patients); 3) follow-up by GPs and surgeons available; 4) able to provide informed consent. | 1) rectal tumours (current practice for rectal cancer follow-up requires regular sigmoidoscopy which would not be undertaken by many GPs). 2) significant polyps discovered at initial colonoscopy (or at subsequent completion colonoscopy) that indicated increased frequency of colonoscopic monitoring. 3) any other condition that warranted increased intensity of surveillance with respect of colon cancer follow-up. | 12 months |

**Table S2.** Continuity of care dimensions in the intervention group.

| **Author, year** | **Mobile health (telephone, web, app etc.)** | **Healthcare providers** | **Need assessment** | **Relational continuity** | **Informational continuity** | **Management continuity** |
| --- | --- | --- | --- | --- | --- | --- |
| Aubin, 2021 | Yes | Nurse navigators, family physician (FP), oncologist | Nurse navigators assess patients' needs | 1) Systematic recommendations to patients to see their FP within the first 3 months after diagnosis. 2) FP is on a 24-hour-a-day, 7-days-a-week on-call system. | 1) A 1-page standardized summary faxed to the FP every 3 months, which included information on lung cancer, patient functional status, prognosis, treatment plan, oncologist recommendations, and nurse navigator contact details; 2) Systematic fax transmissions from the FP to the oncology team of patient information resulting from FP visits. | 1) The research nurse offered help to organize the appointment, and subsequent appointments were arranged between the FP and the patient. 2) the research nurse was instructed to schedule priority access to FPs for patients with minor problems unrelated to cancer if patients were unable to do so. |
| Augestad, 2013 | No | GP, oncologist | NA | 1) replace specialist appointment with patients' GP; 2) relevant phone numbers and contact information were given. | 1) Information was given to the GP about surgery, any complications, Dukes’ staging, time and location of chemotherapy (for Dukes’ C patients), and risk of recurrence. 2) Patient decision-support pamphlet; 3) GP decision-support pamphlet | 1) Patients allocated to GP follow-up could be referred back to any surgical clinic at any time during the study period. 2) In case of questions regarding the follow-up, relevant contact information was given. |
| Bergholdt, 2012&2013 | Yes | GP, rehabilitation coordinators, nurse with oncological experiences | Interviews conducted by rehabilitation coordinator | 1) Patients were advised to consult their GP during treatment and after discharge; 2) GP was encouraged to contact the patient to facilitate a rehabilitation process | The patient’s GP was informed about the patient’s actual problems and needs for rehabilitation by email /phone. | NA |
| Canny, 2022 | No | hospital clinicians, GPs, and primary care teams. | NA | The personal letter was developed with a patient-public involvement (PPI) group and signed by their oncologist to help them make appointment. | General proactive received the same documents plus professional information about ACP, a copy of the NHS Scotland "red-map" communication framework, and a request to consider starting ACP and complete an electronic ACP plan. | NA |
| Emery, 2016 | Yes | GP, specialist | Screened patients for distress using the Distress Thermometer and unmet needs using a prostate cancer-specific problem checklist. | 1) two of the routine hospital visits during the first 12 months of follow-up were replaced by GP visits (at 6 and 9 months); 2) an additional GP visits shortly after the completion of their treatment for prostate cancer was intended to reengaged their patients with his GP. | 1. structured systematic communication (SCP); 2. GP clinical management guidelines and local resources; 3. patient information resources about prostate cancer and treatment side-effects | A register and recall system to prompt the participant and his GP about follow-up appointments |
| Ezendam, 2014 & Nicolaije, 2015 & Rooij, 2017 & Jeppesen, 2017 (ROGY care) | Yes | PCPs, gynaecologists, oncology nurses | NA | NA | Oncology providers (i.e., medical specialists and oncology nurse) in the SCP-care arm were instructed to provide an SCP after initial surgery, not only to the patient but to also send a copy of the SCP to the patient’s PCP. | NA |
| Fethney, 2024 | No | Community nurses (CNs), GP, cancer center staff | CNs used the Chemotherapy Symptom Assessment Scale (C-SAS) to assess patient | NA | 1) Education program to provide CN with relevant cancer cancer and treatment updates, clinical protocols, managing side effects, clinical assessment, communication skills, patient education, treatment and referral pathways inclusive of triage category; 2) Faxed through to the relevant cancer centre and patient’s GP, if participating | 1) Care was shared between CNs, cancer centre staff, participating GPs and any other services/ health professionals referred to by the CN. 2) CNs were provided with contact details for relevant cancer centre staff, if required. 3) Researchers held regular meetings with participating CN centre staff to provide updates and discuss issues arising across all centres. |
| Grunfeld., 2006 | No | Family physicians, oncologist | NA | 1)Provide support and counselling to patients and her family; 2) For patients in the FP group, if a surgeon had been involved in the patient’s follow-up care, that follow-up was also transferred to the FP. | Family physicians were provided with a one-page guideline on follow-up. | Family physicians were instructed to refer patients back to the cancer center if a recurrence or new primary breast cancer developed. |
| Holtedahl, 2005 | No | GP, physician | NA | 1)30-minute consultation with the patients GP, and let them to talk about experiences as a cancer patient. And to tell the patient explicitly that she or he would be welcome to contact the GP whenever there was a question or a problem related to their disease. 2) physician-initiated consultation 6 months later. | NA | NA |
| Jefford, 2023 | Yes | Specialist, PCP | NA | 1) Followed the same model as usual care but replaced the specialist appointments at 3 and 9 months with a GP appointment; 2) Added an additional GP appointment at 2–6 weeks following the end of treatment to re-establish contact and discuss follow-up care. | 1) a tailored survivorship care plan, the ‘Living Well after Cancer’ booklet produced by Cancer Council (a national cancer charity) and a DVD titled ‘Just Take It Day to Day,’41 which was later provided as a weblink. 2) A ‘common issues and concerns’ checklist was administered prior to GP clinic attendance to assist with identification of individual needs. 3) The patient’s GP also received management guidelines detailing common issues experienced by CRC survivors and how to manage these, as well as details on how best to contact the specialist treating team for advice or if recurrence was suspected. 4) A survivorship care plan was pre- pared by the research team, approved by the treating specialist and provided to both the patient and their GP. | Both patients and GPs received a reminder letter about up- coming follow-up appointments, with GPs further reminded to provide information on patient progress and to copy pathology results to the hospital-based team. |
| Jiwa, 2013 | Yes | GP, breast care nurse, other members working only in the community including a dietician, psychologist, sexologist, physiotherapist, occupational therapist and a general medical practitioner. | Self-administered care needs assessment on physical, psychological, social and be interviewed by a breast care nurse. | Telephone follow-up to assess patient referral and check if they have further concerns | Need assessment by nurse working with the patient's hospital based, and the team made recommendations to the GP as a care plan summary to GP. | 1) use electronic patient record and endorse by every member of the team, available by GP; 2) Members of the multidisciplinary team discussed the audio-recorded interviews within 2 weeks., team together to discussed up to four patients in a 2h meeting, care nurse summarizes patient need. GP agreed to this process and was involved in the subsequent care plan. 3) team members are invited to comment on the plan prepared for GP. 4) Three months later their care plan had been delivered to the GP each participant was contacted by telephone to assess if she had been referred for further care and if she still had concerns or problems as per the schedule. |
| Johansson, 2001 | Yes | Home-care nurses, GP, specialist | By interview, dietary assessment | 1) All patients were followed regularly, and the next interview was scheduled 2–3 months later. (By dietician) 2) scheduled interview with dietician, and contact with project psychologist. | 1) GPs and home-care nurses received copies of the medical record each time the patient was discharged from hospital, or had visited a specialist outpatient clinic. 2) Education in cancer care was arranged during the course of the trial. Home-care nurses and GPs were educated in diagnostics and treatments of the cancer diagnoses, as well as in pain, nausea and diet management, psychosocial support and care in the final stage of life. (12 seminars) | 1) GP was also informed about the cancer diagnosis and the referral to the home- care nurse; 2) regular supervision by an oncology team including a dietician, psychologist, physiotherapist, urotherapist and a specialist nurse; nurses were invited to participate in open supervision groups regularly. 3) all nurses had the possibility to contact the oncology team, during the day Monday to Friday every week during the project period. |
| Johnson, 2015 | Yes | PCPs, specialist, haematologists and medical oncologists, a project coordinator (experienced clinical trials nurse). | NA | Patients were asked to see their PCP after each chemotherapy treatment. ‘Built-in’ visits to GP 5-7 days after each cycle of chemotherapy. | 1) Patient given Patient Held Record (PHR) containing individualised Treatment Roadma; 2) Patient given Side effect management sheets. They are advised that these will be sent to their GP also but recommended that they keep them with the PHR for other health providers to view who may be unfamiliar with the side effects of their treatment. 3) GP is faxed treatment Roadmap, this is then posted with side effect management sheets, a list of contact numbers and information on the project. 4) patient is instructed to take PHR to these and have the GP write in the communications pages. | Patient is asked to bring PHR to SCGH visits and ask medical staff to write in communications pages to facilitate communication and shared care between GP and tertiary setting. |
| Jordhøy, 2000&2001 | No | GP, the community nurse, a consultant nurse, physician in the Palliative Medicine Unit. | Joint meeting with patient, the informal caregiver, the GP, and the community nurse to assess patients' needs and predefined minimum standards. | The patients' GP and community nurse were defined as the main professional caregivers; 2. Follow-up consultations by the GP and the community nurse were arranged according to the patients’ needs and predefined minimum standards. | 1) Treatment plan was set up in joint meetings (PMU and community services); 2) An educational program for the community professionals included bedside training and 6 to 12 hours of lectures every 6 months. | 1. The PMU consultant team participated in the inpatient care, handled the PMU outpatient clinic, coordinated the follow-up; 2. The PMU consultant team was available to the community staff for supervision and advice and to join visits in the patient’s home. |
| Kvale, 2016 | Yes | Coach, PCP, nurses, nurse practitioners, and trained community volunteers | Used motivational interviewing (MI) techniques to engage patients in the development of a patient-owned SCP | NA | Use of a patient-centered health record to guide the transition, PCP and specialist follow-up, medication management, and patient/caregiver understanding of “red flags” that might indicate worsening of an underlying condition. | 1. use of a patient-centered health record to guide the transition, PCP and specialist follow-up, medication management, and patient/caregiver understanding of “red flags” that might indicate worsening of an underlying condition. 2. “Red flags” for seeking help are reviewed, and the survivor and coach work through which member of the health care team is the appropriate contact for the red flags discussed. |
| Luker, 2000 | No | Breast specialist consultant surgeon, a researcher with expertise in the field of breast cancer and three breast care nurses | NA | The women would be given the cards while in secondary care, by a breast care nurse or clinic nurse, and asked to take them to the PHCT; Women would **not be** instructed to attend the PHCT for follow-up as such and it was **not** the intention to replace the current hospital follow-up regime. | 1) A series of 11 information cards were developed and included a booklet/card on each of the subjects to assist the PHCT in meeting the information needs of women with breast cancer. 2)Women were given cards relevant to their treatment and the number and type of cards given to each woman was determined by the treatment received. | NA |
| Mayer, 2016 | Yes | Oncology NP, PCP | NA | 1) A clinic staff member then scheduled a PCP visit for the survivor; 2) Talking points also suggested discussing any plans for non–cancer-related health care and health promotion, discussing any residual side effects from treatment that may still be present. | 1) PCP visit arm received a printed copy of their SCP; All PCPs were sent the final SCP electronically (if accessible within the electronic health record) or by mail; 2) Talking points developed for this visit were developed and sent to the PCP along with the SCP to encourage reviewing the SCP. 3) two websites about cancer survivorship care for PCPs. | clear what type of care the PCP would provide, and when and for what type of concerns the survivor should contact the PCP or the oncologist. |
| Mitchell, 2008 | Yes | GPs and a specialist team, family physician, or domiciliary nurses | NA | NA | The case conference negotiating a treatment plan for the patient, with the GP playing an active part. | 1. case conference was conducted by teleconference, with the GP phoning in to a routine specialist team meeting, and telephone communication between family physician and specialist, or domiciliary nurses present at specialist team meetings acting as an intermediary. 2. those whose GP was encouraged to participate in a case conference with the specialist team (as near as practicable to the referral date, target within 3 weeks) |
| Nielsen, 2003 | No | GPs, doctors, nurses | NA | Patients were encouraged to contact their GP when facing problems, they assumed could be solved in this setting | 1) discharge summary letters following predefined guidelines, with the information on disease, treatment, and general information; 2) patients received oral as well as written information about the information package to their GP | 1) Names and phone numbers of doctors and nurses responsible for the patient were attached to the discharge summary letter to the GPs |
| Perfors, 2022 | Yes | Home care oncology nurses (HON, a part of primary care), GP, medical doctors | NA | 1) Intervention patients were advised to make a TOC (Time out consultation) appointment with their GP; 2) Home visit or telephone contact by home care oncology nurse, in collaboration with GP. | 1) The GP receives information on the diagnosis and treatment plan by phone or by mail; 2) The surgeon providing the woman’s post-operative outpatient review after the MDT meeting; 3) patients in the intervention group were offered structured guidance from primary care | 1) During the TOC, joint guidance by the GP and a HON was offered to the patient. The HON reported the condition of the patient and required actions to the GP. 2) The hospital was also informed by the HON, in case supportive care was started based on the HON’s consultations (e.g., consultation of a psychologist, physiotherapist or dietician) or when treatment-specific questions arose. |
| Rio, 2017 | No | Surgeon, GP, senior clinical nurse, hospital nurse | NA | Women were instructed to see their GP in approximately one month to discuss the care plan. | 1) A letter detailing the findings of the MDT meeting and a woman’s eligibility were sent to a woman’s GP Care plans were then ratified by the Head of Gynaecology Oncology Unit, with a copy sent to both the woman and her GP. | 1) The GP was subsequently called by the nurse to: ensure that the care plan was received; ensure the GP would provide follow-up care; 2) reinforce the schedule of visits and hospital re-referral and support pathways; and address any queries or concerns. |
| Taylor, 2019 | No | cancer nurse coordinator (CNC), haematologist, GP | NA | Participants were encouraged to discuss the follow-up recommendations with their GP. | 1) The resource pack contents were explained and information on how to access support provided; 2) Copies of the completed SCPTS were distributed to the participant, their GP and their hospital record. | NA |
| Wattchow, 2006 | No | Surgeon, GP | NA | NA | Follow-up guidance, based on current clinical practice and guidance was provided, and inserted into either the patient’s GP or surgeon/hospital records. | Patients allocated to ‘GP-led’ follow-up could be referred back to surgical clinics at any point in the study. |

NA, not available.

**Table S3.** Subgroup analysis on the quality of life.

| **Study subgroup characteristics** | **Measures (N)** | **SMD (95%)** | ***P* for interaction** |
| --- | --- | --- | --- |
| **Overall** | 23 | 0.01 (-0.04,0.05) | - |
| **Continuity of care dimensions** |  |  | 0.959 |
| All | 11 | 0.01 (-0.07, 0.08) |  |
| Partial | 12 | 0.00 (-0.07, 0.08) |  |
| **Relational continuity*** |  |  |  |
| **Informational continuity** |  |  | 0.447 |
| Yes | 21 | 0.01 (-0.05, 0.07) |  |
| No | 2 | -0.02 (-0.41, 0.27) |  |
| **Management continuity** |  |  | 0.959 |
| Yes | 11 | 0.01 (-0.07, 0.08) |  |
| No | 12 | 0.00 (-0.07, 0.08) |  |
| **Cancer types** |  |  | 0.868 |
| Colorectal/breast/prostate cancers | 8 | 0.01 (-0.11, 0.13) |  |
| Other cancer types | 15 | 0.00 (-0.05, 0.06) |  |
| **Need assessment** |  |  | 0.822 |
| Yes | 3 | 0.01 (-0.12, 0.14) |  |
| No | 20 | 0.00 (-0.07, 0.07) |  |
| **Multidisciplinary team involved** |  |  | 0.868 |
| Yes | 15 | 0.00 (-0.05, 0.06) |  |
| No | 8 | 0.01 (-0.11, 0.13) |  |
| **Mobile Health** |  |  | 0.384 |
| Yes | 5 | -0.01 (-0.11, 0.08) |  |
| No | 18 | 0.03 (-0.05, 0.10) |  |
| **Follow-up length** |  |  | 0.771 |
| No more than 3 months | 6 | -0.01 (-0.13, 0.15) |  |
| 3-6 months | 6 | -0.00 (-0.13, 0.12) |  |
| 6-12 months | 7 | 0.05 (-0.10, 0.19) |  |
| More than 12 months | 4 | -0.02 (-0.10, 0.07) |  |
| **Measurements** |  |  | 0.428 |
| EORTC QLQ C-30 | 11 | 0.00 (-0.05, 0.06) |  |
| EQ-5D index | 6 | -0.01 (-0.33,0.31) |  |
| EQ-5D VAS | 6 | 0.07 (-0.04, 0.17) |  |

*All included study involved relational continuity components

EORTC QLQ C-30: European Organization for Research and Treatment of Cancer Core Quality of Life questionnaire; EQ-5D, EuroQol- 5 Dimension.


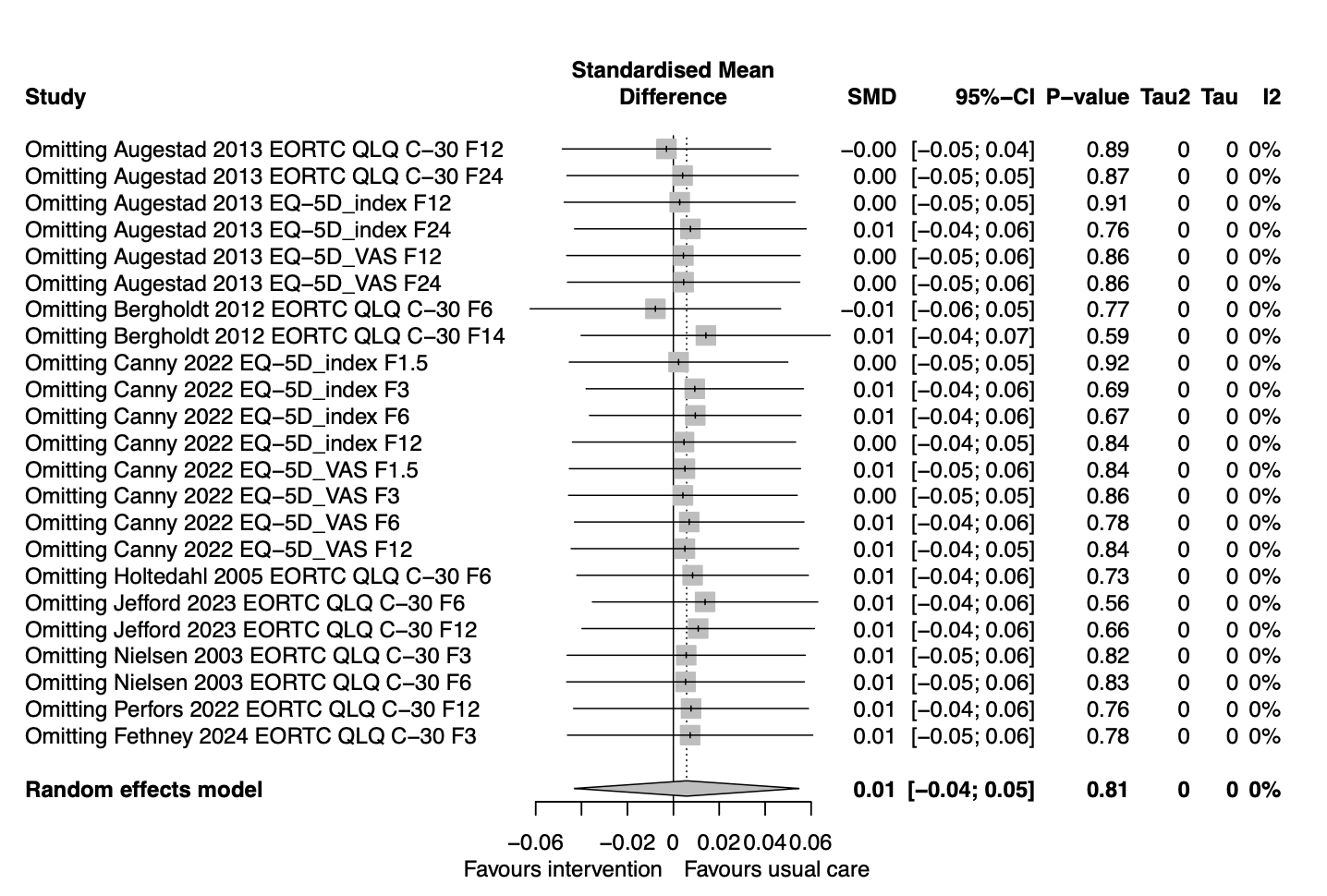


**Figure S1.** Sensitivity analysis by the leave-one-out method on quality of life.


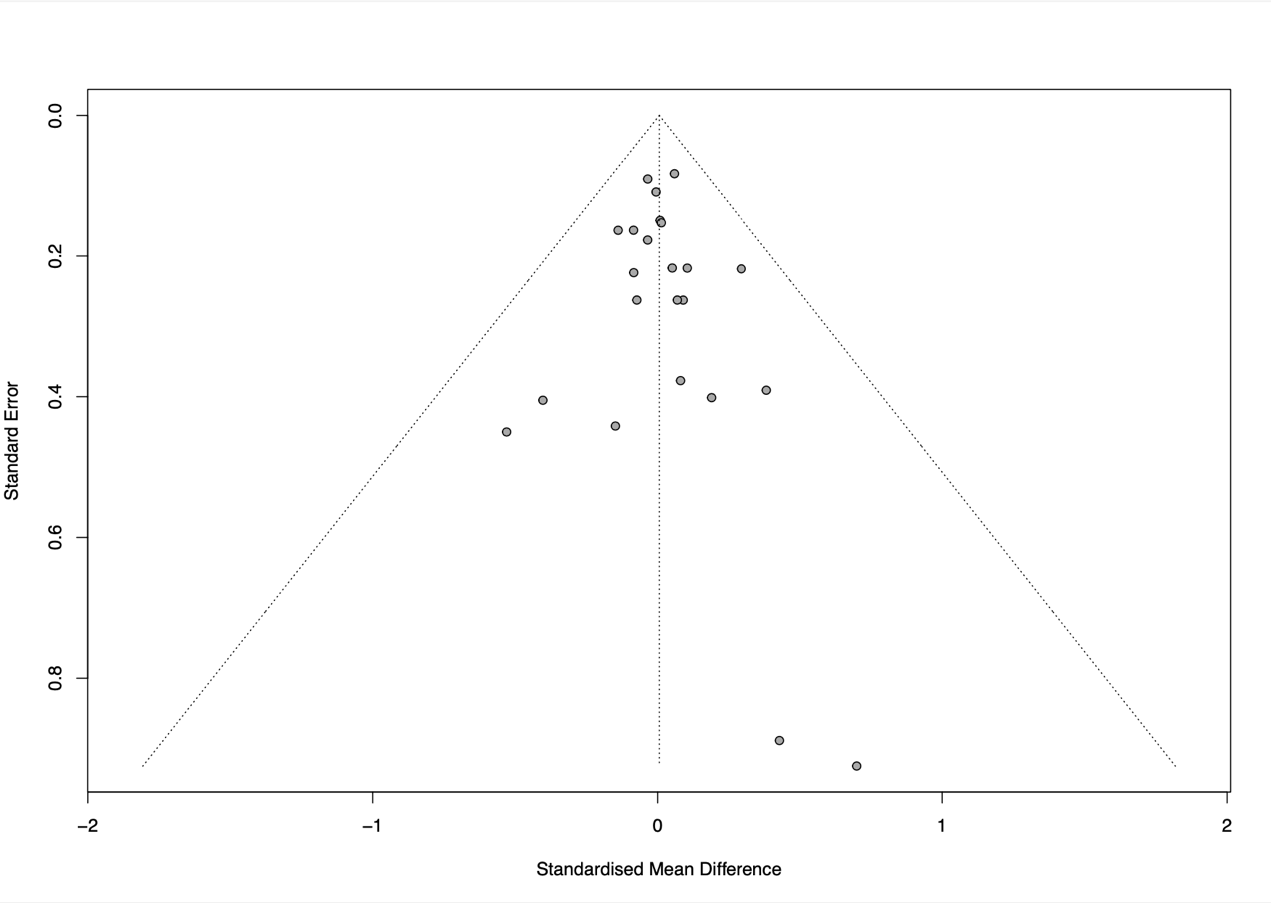


**Figure S2.** Funnel plot assessing publication bias for the quality-of-life outcomes.


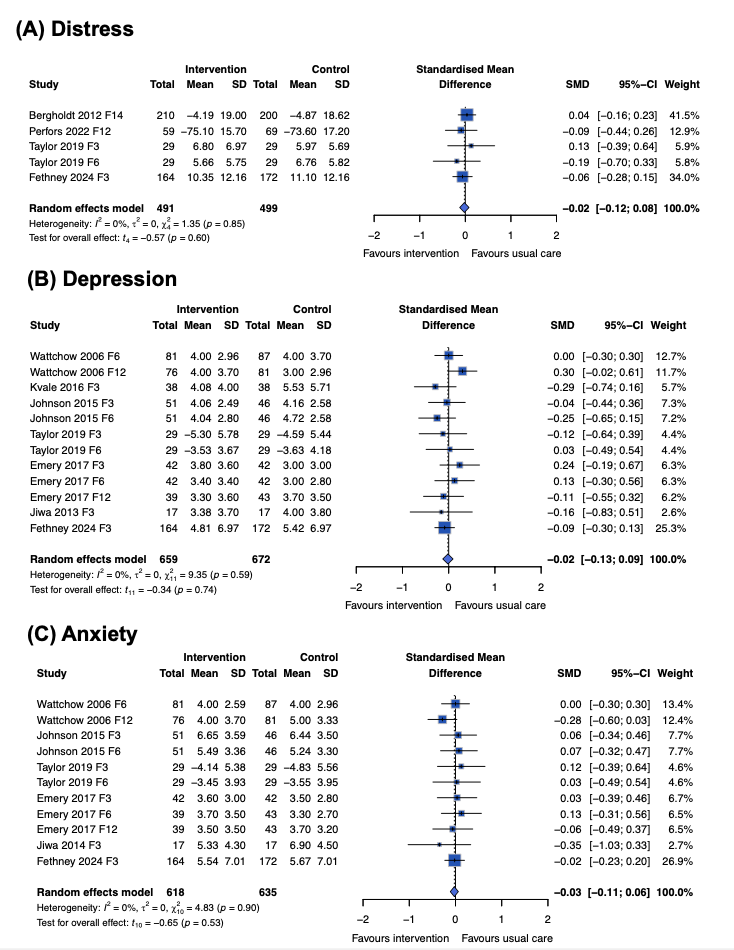


**Figure S3.** Forest plot of pooled means for **(A)** distress; **(B)** depression; **(C)** anxiety. Number following F in the study column denotes the length of follow-up months.

**Figure S4.** Forest plot of pooled means for perceived **(A)** continuity of care; **(B)** satisfaction. Number following F in the study column denotes the length of follow-up months.

**
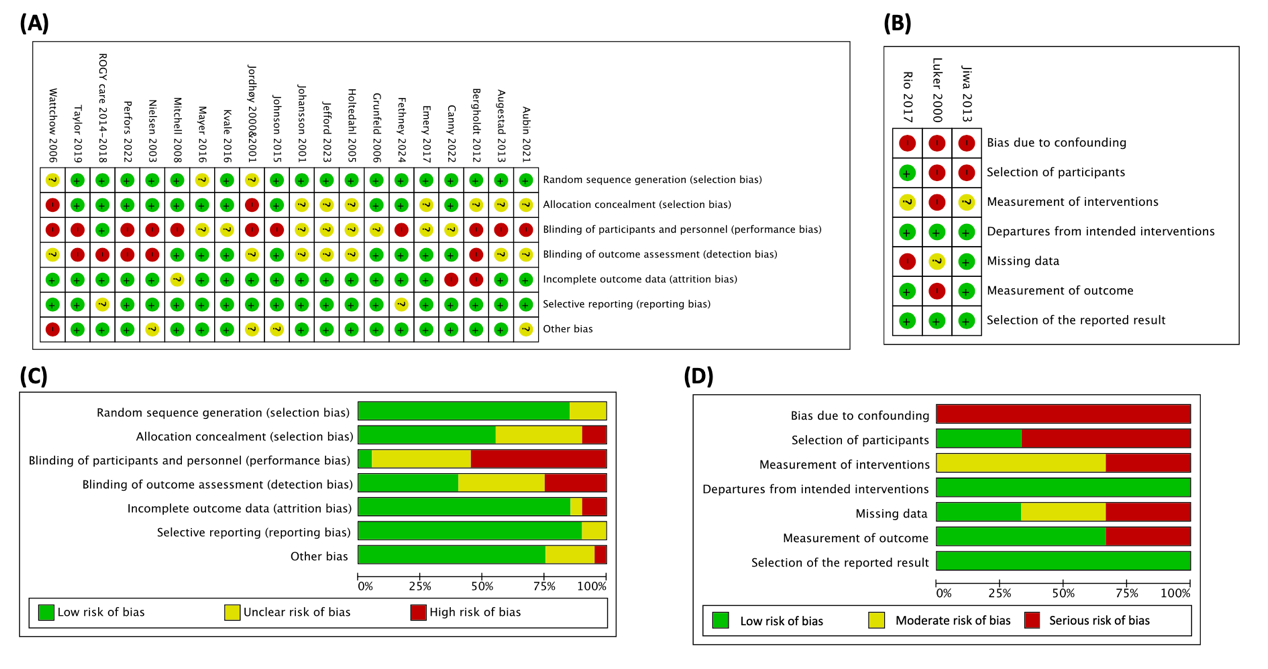
**

**Figure S5.** Results of risk of bias presented by each study for **(A)** randomize control trials; **(B)** non-randomized control trials; presented as percentage across all included studies of **(C)** randomize control trials, and **(D)** non-randomized control trials.
